# Twitter Use by Academic Nuclear Medicine Programs: Pilot Content Analysis Study

**DOI:** 10.2196/24448

**Published:** 2021-11-08

**Authors:** Ananya Panda, Akash Sharma, Ayca Dundar, Ann Packard, Lee Aase, Amy Kotsenas, Ayse Tuba Kendi

**Affiliations:** 1 Department of Radiology Mayo Clinic Rochester, MN United States; 2 Department of Radiology Mayo Clinic Jacksonville, FL United States; 3 Social Media Network Mayo Clinic Rochester, MN United States

**Keywords:** social media, Twitter, radiology, nuclear medicine, nuclear radiology, social network, medical education, networking

## Abstract

**Background:**

There is scant insight into the presence of nuclear medicine (NM) and nuclear radiology (NR) programs on social media.

**Objective:**

Our purpose was to assess Twitter engagement by academic NM/NR programs in the United States.

**Methods:**

We measured Twitter engagement by the academic NM/NR community, accounting for various NM/NR certification pathways. The Twitter presence of NM/NR programs at both the department and program director level was identified. Tweets by programs were cross-referenced against potential high-yield NM- or NR-related hashtags, and tabulated at a binary level. A brief survey was done to identify obstacles and benefits to Twitter use by academic NM/NR faculty.

**Results:**

For 2019-2020, 88 unique programs in the United States offered NM/NR certification pathways. Of these, 52% (46/88) had Twitter accounts and 24% (21/88) had at least one post related to NM/NR. Only three radiology departments had unique Twitter accounts for the NM/molecular imaging division. Of the other 103 diagnostic radiology residency programs, only 16% (16/103) had a presence on Twitter and 5% (5/103) had tweets about NM/NR. Only 9% (8/88) of NM/NR program directors were on Twitter, and three program directors tweeted about NM/NR. The survey revealed a lack of clarity and resources around using Twitter, although respondents acknowledged the perceived value of Twitter engagement for attracting younger trainees.

**Conclusions:**

Currently, there is minimal Twitter engagement by the academic NM/NR community. The perceived value of Twitter engagement is counterbalanced by identifiable obstacles. Given radiologists’ overall positive views of social media’s usefulness, scant social media engagement by the NM community may represent a missed opportunity. More Twitter engagement and further research by trainees and colleagues should be encouraged, as well as the streamlined use of unique hashtags.

## Introduction

Social media use among adults in the United States has increased substantially over the last decade, with the percentage of people who use at least one social media account doubling from 36% in 2009 to 72% in 2019 [1]. As with the general population trends, there has been a burgeoning use of social media by health care professionals, including radiologists. Although early research on the use of social media focused on physician-patient communication, the same enthusiasm is now developing for professional networking, education, and peer engagement. In recent surveys, 60%-85% of radiologist respondents acknowledged using social media for a mix of professional and personal purposes [2,3]. Specifically, social media is more popular among medical students and radiology trainees; they aim to use it for education, information gathering, informal engagement, and mentorship opportunities with experienced faculty members [4]. Of all the social media platforms offering unique features to engage with a wider audience online [5], Twitter is particularly popular for professional use as it allows real-time, multilateral online conversations, and sharing of a wide range of content [6]. Both program directors as individuals and academic radiology departments as group entities are increasingly receptive to Twitter as an effective medium for academic activity; this includes radiology education, recruitment of future faculty and trainees, peer networking, and opportunities for collaboration and engagement on the basis of similar interests [7-9].

As a specialty, nuclear medicine (NM) and Molecular Imaging is complex as there are several pathways to achieve posttraining certification in NM or nuclear radiology (NR). Physicians and scientists—both those trained in radiology and those not trained in radiology—pursue the various training pathway options in the United States [10]. Currently, training pathways include the following: (1) a traditional NM residency program embedded within the radiology department (~39 programs as of 2019), (2) a 1-year NR fellowship after diagnostic radiology training, and (3) a dual pathway comprising 16 months of NR training integrated into a diagnostic radiology (DR) or intervention radiology residency (~50 DR programs and 1 intervention radiology program as of 2019). Despite attempts to increase the number of physicians entering NM/NR by reforming training requirements, a recent survey found a lack of exposure to NM and NR among medical students and early radiology residents [11], with reinforced calls to improve outreach efforts to medical students and radiology residents [11,12].

Although these two developments as described are separate, we propose that social media may represent an opportunity and serve as a resource that the academic NM/NR community can use for outreach efforts aimed at attracting future trainees. However, to our knowledge, there is very little information on the presence of NM and NR programs on social media, specifically Twitter.

Social media analytics tools such as Symplur allow codifying of data through ontology hashtags on Twitter [13]. These ontology hashtags aid in the topical organization of tweets, thereby channeling overwhelming amounts of data into more relevant and consumable data streams. The radiology tag ontology project was devised in 2015 as an initiative specifically aimed at codifying a list of radiology-related hashtags that people can use to tag social media content so that it may be discovered by others with similar interests [13].

Our purpose was to assess Twitter engagement by academic NM/NR programs in the United States, and to further characterize the value proposition of social media use in this specialty.

## Methods

Institutional Review Board approval was deemed not necessary for this study as it is an internet-based study accessing publicly available information. We compiled a composite list of programs that offered NM residency, NR fellowships, and dual DR/NR pathways and radiology residencies for 2019-2020, obtained from the American Board of Medical Specialties, the American Board of Nuclear Medicine (ABNM), the Accreditation Council for Graduate Medical Education, and the Electronic Residency Application System [14-16]. If a program offered more than one training pathway, it was counted only once and was grouped into NM residency, NR fellowship, and DR/NR residency in decreasing hierarchical order. The list of program directors for NM residencies, fellowship directors for NR fellowships, program directors for DR programs, and NR division chief preceptors for the 16-month DR/NR pathway was obtained through the same online sources, as well as the American Board of Radiology (ABR); we also used individual radiology program websites for any missing data [15,16].

In December 2019, a manual search for Twitter handles was done for each of these radiology programs, followed by consensus reconciliation by a radiology fellow and a radiology resident. The search process was iterative, using the “Search,” “Top,” and “People” features of Twitter. The Twitter presence of a training program was considered positive if the program had a Twitter handle (account) for the radiology department or radiology residents. If a program had additional Twitter handles specifically for its NM/NR division, it was noted separately. A manual search using the “Search” and “People” features was also done to identify individual Twitter handles of program directors for NM residencies, fellowship directors for NR fellowships, program directors for DR programs, and/or NR division chairs for those programs only offering DR/NR pathways. These individuals are collectively referred to as program directors throughout. A program director Twitter presence was considered positive if any of the above individuals meeting the definition of a program director had an individual Twitter account. For general radiology residency programs that do not offer NM/NR training pathways but have a NM/NR division, the NM division chiefs were considered as surrogates for a program director to measure Twitter presence (ie, if the division chair had a Twitter handle, it was considered equivalent to program director Twitter presence, even if the division did not offer NM or NR certification). The search was done by two people to improve yield and minimize data deletion.

We then conducted a search cross-matching all potential NM- or NR-related hashtags (Table 1) for each program with a Twitter handle and for program directors with Twitter accounts. These hashtags were selected to broadly encompass the different aspects of this specialty; we also included hashtags used by the Society of Nuclear Medicine and Molecular Imaging (SNMMI) in its tweets. A post by a program with any of these tags between January-December 2019 was considered as positive for NM- or NR-related Twitter activity, irrespective of the number of tweets. Similarly, program director Twitter activity was defined as at least one NM- or NR-related tweet by the program director in the year 2019. Although this search was done for all potential combinations of hashtags related to NM/NR, #nucmed and #molrad are the only radiology ontology hashtags currently catalogued by the social media analytic tool Symplur [13]. A further subanalysis of individual tweets for the month of December 2019 was done using Symplur and the contents of tweets were categorized as related to radiology education, patient education, departmental promotion, conference talks or lectures, research, and peer networking. Lastly, we conducted a brief survey to assess trends with respect to factors that would obstruct or facilitate NM/NR physicians’ engagement on social media. A SurveyMonkey survey was sent to all email addresses for NM/NR programs available through the Nuclear Medicine Program Directors Association (NMPDA). In total, approximately 45 physicians or program staff were contacted. The survey was anonymized but it allowed the respondents to self-identify as a program director, associate program director, program faculty, or program coordinator. The survey questions are enumerated in Multimedia Appendix 1.

**Table 1:** List of potential nuclear medicine and nuclear radiology hashtags queried to measure the Twitter presence of academic radiology programs.

Twitter hashtag	Reason selected
#nucmed	Hashtag for nuclear medicine mentioned in the radiology ontology project
#molrad	Hashtag for molecular imaging mentioned in radiology ontology project
#nuclearmedicine	Full expansion of subspecialty
#nuclearradiology	Full expansion of subspecialty
#molecularimaging	Full expansion of subspecialty
#petct	Popular hybrid imaging modality
#petmri	Upcoming hybrid imaging modality
#precisionmedicine	Used by Society of Nuclear Medicine and Molecular Imaging in its tweets to promote the field
#precisionimaging	Used by Society of Nuclear Medicine and Molecular Imaging in its outreach tweets to promote the field
#FOAM #nucmed	Hashtag used to promote Free and Open Access to Medical Education (FOAM) on Twitter, considered synonymous with education
#FOAMrad #nucmed	Hashtag used to promote Free and Open Access Medical Education, considered synonymous with radiology trainee education

## Results

For 2019-2020, 39 unique programs were included under NM residency, 15 under NR fellowship, and 34 under the DR/NR category. The 34 programs in the DR/NR category only offered the DR/NR pathway without NR fellowships. Thus, 88 radiology programs offered training pathways to certification in NM or NR. Of the other 103 radiology residencies, the residents may have only been exposed to NM rotations as part of their radiology residency, without a formal pathway for certification. The results for Twitter engagement by radiology programs and program directors are summarized in Table 2.

**Table 2:** Summary of Twitter activity related to nuclear medicine or nuclear radiology for radiology programs in 2019-2020.

Activity	NM^a^ residency (n=39), n (%)	NR^b^ fellowship (n=15), n (%)	Dual DR^c^/NR pathway (n=34), n (%)	Other radiology residencies (n=103), n (%)
Radiology Twitter handle	24 (62)	8 (53)	14 (41)	16 (16)
NM- or NR-specific Twitter handle	3 (8)	0 (0)	0 (0)	0 (0)
Radiology handles tweeting about NM- or NR-related content	13 (33)	4 (27)	4 (9)	5 (5)
Program directors with Twitter handles	3 (8)	4 (27)	0 (0)	0 (0)
Program directors tweeting about NM- or NR-related content	2 (5)	1 (7)	0 (0)	0 (0)

^a^NM: nuclear medicine.

^b^NR: nuclear radiology.

^c^DR: diagnostic radiology.

Out of all programs offering NM/NR training pathways, 46/88 (52%) had radiology accounts on Twitter but only 3/88 (4%) had an exclusive Twitter handle for NM or NR. Of these 88 programs, 21/88 (24%) had at least one tweet related to NM/NR. However, programs offering only DR/NR pathways were less likely to have tweeted about NM- or NR-related content (4/34, 9%) than NM residencies (13/39, 33%) and NR fellowships (4/15, 27%; Table 1). Of the other 103 radiology residencies, 16/103 (16%) had Twitter handles for radiology, with only 5/103 (5%) tweeting about NM- or NR-related content. The program directors’ presence was also low as only 7/88 (8%) NM/NR program directors had Twitter handles. None of the NM division chiefs from the other 103 radiology residencies had an identifiable Twitter handle. Only 3/191 (2%) of program directors actively tweeted about NM/NR (Table 2).

A content-based subanalysis of NM- and NR-related tweets in December 2019 cross-referenced against hashtags revealed 6 primary tweets by 3 programs for #nucmed, 5 of which were related to NM talks at the annual meeting of the Radiology Society of North America (RSNA), while the remaining tweet was related to department promotion. There were no tweets for the radiology ontology hashtag #molrad but there were 6 tweets/retweets by 6 departments for #molecularimaging, related to RSNA talks and department lectures (n=5) and department promotion (n=1). There were no tweets by academic NM/NR programs with other hashtags such as #precisionmedicine, #precisionimaging, #nuclearmedicine, #FOAMRad #nucmed, and #FOAMed #nucmed. There were also no tweets by NM/NR programs related to radiology education, patient education, research activities, and peer networking.

The brief survey sent to NM/NR faculty and staff via NMPDA contacts further characterized the status of social media use. Overall, one-third of the people contacted (15/45) responded to the survey. The majority of respondents (12/15, 80%) confirmed that they did not have a Twitter handle for their role in their training program; only 3 (20%) had Twitter handles. The majority (11/15, 73%) also confirmed that their programs did not have a unique Twitter handle for their radiology department or NM/NR divisions; only 4 respondents said that their programs had unique Twitter handles. When forced to rank the deterrents to engagement on Twitter, all the listed issues were considered relevant, with no dominant hurdle (Table 3). Despite these perceived hurdles, the 15 respondents thought that the most compelling reason for social media use may be the perceived value of social media for engaging the younger generation of trainees (Table 4).

**Table 3:** Forced ranking of reasons not to use Twitter.

Reason	Score (1=lowest, 5=highest)
Limited resources (assistance from staff/time to do the work)	3.3
Lack of clarity of value of social media in education	3.6
Lack of expertise among the program director, associate program director, and coordinator	3.4
Another Twitter handle already provides some coverage for this training program	2.6
Negative prior social media experience in a professional setting	2.7

**Table 4:** Forced ranking of reasons for using Twitter.

Reason	Score (1=lowest, 5=highest)
Perceived value of social media for the younger generation of trainees	4.6
Free marketing	2
Effective way to highlight your training program	3.2
Networking with other programs/organizations	2.2
Follow trends in education in your subspecialty	2.9

## Discussion

This study aimed to measure Twitter engagement by academic NM and NR programs in the United States during 2019-2020. Despite 88 programs offering potential pathways to ABNM or ABR NR subspecialty certification, only 3 programs had exclusive handles for the NM division. Although just over half of the 88 programs (n=46, 52%) had a Twitter handle for the broader radiology department, less than one-fourth (n=21, 24%) of all programs tweeted about content related to NM or NR in 2019. The programs offering only DR/NR pathways tweeted about NM- and NR-related content much less than other NM residencies and NR fellowships. Other radiology residencies without NM or NR training pathways also had low Twitter presence (16/103, 16%) and lower Twitter activity related to NM or NR content. Additionally, only 8% (7/88) of NM/NR programs had a DR/NR program director available on Twitter. These findings indicate that there is a substantial missed opportunity for reaching out to or networking with future trainees, a group that has been shown to be open to using social media in their professional development. Although it may not always be possible—or may even be against existing institutional policies—to allow separate Twitter handles for individual divisions of a department (eg, NM/NR), the overall paucity of Twitter handles for academic NM/NR programs as well as general radiology programs is somewhat remarkable.

The findings of this study are particularly relevant given recent reports of the increasing workforce demand for NM professionals. When juxtaposed against the lack of early exposure and awareness of NM/NR training among medical students and even radiology residents [11,12], it seems NM as a field is not availing itself of a potential method to engage the increasing number of medical students (#medstudents, #medstudentTwitter) and future radiology residents (#futureradres) who are turning to Twitter in an effort to gather information about residency programs [17]. Thus, there is an inherent need for NM/NR academic programs to improve their presence on social media sites such as Twitter and aim for greater online visibility of their own programs. Having more information about NM/NR as a subspecialty and offering engagement opportunities for mentorship, electives, education, and research experiences will not only benefit the current cohort of trainees but also build the foundation for future development of the subspecialty. Ultimately, both will directly benefit patients by addressing the existing and increasing demand for NM/NR physicians.

A brief survey done after the analysis of the preliminary data attempted to elicit which common issues may be hurdles for the NM/NR academic community in increasing their social media presence. Although only one-third of people contacted (15/45) responded to the survey, the results are helpful to highlight both the challenges and the benefits to social media use. The respondents included program directors, associate program directors, faculty members, and program staff. The respondents confirmed our observation that most programs and program directors do not have unique Twitter handles for their programs. When asked to do a forced ranking of potential reasons to not use Twitter, lack of clarity on the value of social media, lack of expertise, and limited resources to engage on social media were commonly cited by the respondents (Table 3). Although lack of resources is a common issue in many academic operations, the lack of expertise with social media and prior personal negative experiences on social media are also deterrents. Despite these hurdles, the 15 respondents believed that social media could be valuable for engaging the younger generation of trainees (Table 4).

Though our findings demonstrate the limited social media presence of academic NM/NR and DR/NR programs, the general trend of increased use of social media in health care suggests that this specialty may yet find value in increased social media engagement. There are some specific steps that can be taken to make social media use more valuable for the NM/NR community in the future: (1) streamlined consensus use of hashtags, (2) co-opt professional societies to lead the hashtag initiative, and (3) continue to study utilization of social media at the group level in NM/NR in the context of general health care–related use and provide ongoing feedback to our professional community.

Future work may consider each of these value propositions. As part of greater Twitter engagement by the academic NM/NR community, streamlining hashtags related to NM/NR (as summarized in Table 1) and more consistent use of these hashtags may promote aggregation of NM/NR content and more efficiently connect people looking for this information [18-22]. Although #nucmed and #molrad have found a place in the radiology ontology, not all tweets related to NM/NR use these hashtags. This may be because the few hashtags listed in the radiology ontology project were not developed by nuclear radiologists, resulting in a lack of awareness of these hashtags among those in the field of radiology. Further, while the SNMMI (@SNM_MI) often uses #nuclearmedicine and #precisionmedicine in tweets, they have not used #nucmed and #molrad. The American College of Nuclear Medicine and its Nuclear Medicine Resident Organization (@nmroacnm) could consider collaborating with other professional societies with wider Twitter impact, such as the American College of Radiology (ACR), in promoting their specialty. Another option could include reaching out to the Association of Program Directors in Radiology (@theAPDR) to advocate for more frequent discussion of NM/NR and DR/NR training pathways online and specifically seeking out opportunities to engage medical students and radiology residents early in their training. NM societies such as the SNMMI should also consider leading the community in increasing social media engagement. Just as the RSNA and the ACR focused substantially on social media at their recent annual meetings [20,23], the SNMMI may consider planning sessions focused on harnessing the potential power of social media both during NM society meetings and for ongoing conversations.

This study has several limitations. First, this is a preliminary study that only evaluated engagement on Twitter; other social media networks were not evaluated. We also did not compare the engagement of academic NM with other related specialties such as medical oncology, radiation oncology, and nuclear cardiology, which comprise the top three specialties allied with NM. However, we assessed the lack of engagement against the backdrop of the broad inherent possibility of potential professional interactions at large. We were only able to perform a content-based subanalysis for one month using the free version of Symplur and our search process was manual. We also did not assess the number of followers each program had, or the retweets or social media influence of tweets themselves. Although social media use is higher among younger trainees and medical students [24], we did not have the means to readily correlate the age of program directors, the size of programs, and the number of trainees enrolled as these data are not freely available. These limitations also highlight the general need for more rigorous analytics on social media use in health care. We only considered tweets and retweets by academic NM/NR programs and did not include tweets by people in private radiology practice, scientists, and industry partners who may have higher levels of engagement on Twitter [25]. However, if any of these tweets were retweeted by a particular academic radiology program, it was considered as positive for Twitter presence for that program. The survey did not have a free-text option to capture other responses. Further studies with a broader scope are needed to address each of these limitations. A survey with a larger sample size and a qualitative or free-text component may help us develop a better understanding of barriers to and reasons for social media use in the NM/NR community.

There is currently very little Twitter engagement by the academic nuclear medicine community. This is adequately corroborated by scan data of Twitter use and our attempt to survey faculty in the discipline. Although there are identifiable obstacles, the responses by NM faculty and staff as well as the general trend of increased social media use among medical students substantially support the perceived value in increasing social media engagement in imaging specialties. A more in-depth investigation in the future may further help us understand the barriers and benefits of social media use, and assess the impact of increased use on trainee recruitment and perceptions. Additionally, the value proposition of streamlining and growing social media engagement with targeted hashtags may be considered to promote the presence of both diagnostic and therapeutic aspects of this subspecialty for practitioners, trainees, and the public.

## Appendix

Multimedia Appendix 1 Survey questions.

## Abbreviations

ABR: American Board of Radiology
ACR: American College of Radiology
DR: diagnostic radiology
NM: nuclear medicine
NMPDA: Nuclear Medicine Program Directors Association
NR: nuclear radiology
RSNA: Radiology Society of North America
SNMMI: Society of Nuclear Medicine and Molecular Imaging
