# Supplementary material for: Twitter Use by Academic Nuclear Medicine Programs: Pilot Content Analysis Study
Source: JMIR Form Res. 2021 Nov 8;5(11):e24448. doi: 10.2196/24448 (PMC8663684; doi:10.2196/24448)
Supplement: Multimedia Appendix 1 [file formative_v5i11e24448_app1.docx]

| **Appendix 1: Survey Questions on Social Media Usage by Nuclear Medicine Training Programs** |
| --- |

Top of Form

1. What is your role in the training program?

Program Director

Associate/Assistant Program Director

Faculty member - Nuclear Medicine

Faculty member - Nuclear Radiology

Program Coordinator

2. Do you have a unique Twitter handle that you use for your role in the training program?

Yes

No

If yes, please provide Twitter handle

3. Does the program have a unique Twitter handle?

Yes

No

If yes, please provide Twitter handle

4. **If you do not use Twitter** as part of your engagement strategy for future trainees, please rank each of the reasons below from 1 to 5 in order of relevance with respect to your program:

- Limited resources (assistance from staff/time to do the work)
- Lack of clarity of value of social media in education
- Lack of expertise among the PD/APD/Coordinator
- Another Twitter handle already provides some coverage for this training program
- Negative prior social media experience in a professional setting

**Question Title**

5. **If you do use Twitter** as part of your engagement strategy for future trainees, please rank the each of the reasons below from 1 to 5 in order of relevance with respect to your program:

- Perceived value of social media for the younger generation of trainees
- Free marketing
- Effective way to highlight your training program
- Networking with other programs/organizations
- Follow trends in education in your sub-specialty

Bottom of Form
